# The Syndemic Approach in relation to Clinical Practice and Research in Psychiatry

**DOI:** 10.17650/2712-7672-2020-1-2-3-6

**Published:** 2020-12-04

**Authors:** Sarah J. Parry, Graham Thornicroft

**Affiliations:** South London and Maudsley NHS Trust, Ladywell Unit; Centre for Global Mental Health and Centre for Implementation Science, Institute of Psychiatry, Psychology and Neuroscience, King's College London

**Keywords:** syndemic, psychiatry, research, science, синдемия, психиатрия, исследования, наука

## Abstract

The syndemic framework goes beyond the concept of comorbidity and considers how diseases interact within their wider environmental context, along with social and political factors, to mutually exacerbate negative outcomes. The syndemic approach enhances the way mental disorders are understood in terms of their aetiology, treatment and prognosis and therefore influences the direction of clinical practice, policy development and research priorities in the field of psychiatry. Using a syndemic framework to develop mental health policy globally can help address the mental health treatment gap in countries where resources are limited. In Russia, identified syndemics have been of particular relevance to mental disorders and further research using a syndemic framework will continue to build upon the strong background of integrated mental healthcare currently provided.

## INTRODUCTION

The syndemic approach is highly relevant to both clinical practice and research in psychiatry. The ways in which mental disorders are understood in terms of their aetiology, treatment and prognosis inevitably has an influence on the direction of policy development, on clinical practice and on research priorities in the field of psychiatry. Using a syndemic approach to understand the mental health context in Russia provides an opportunity to enhance the development of effective policy, services and mental health interventions.

## THE SYNDEMIC APPROACH

The term "syndemic" was first coined by the medical anthropologist Merrill Singer in the 1990s to describe the "SAVA" syndemic of substance abuse, violence and HIV/AIDS in an inner-city population in the USA [1]. A syndemic involves two or more diseases that interact to worsen health outcomes and includes consideration of how the wider environmental context and other socio-economic and political factors contribute over time to mutually exacerbate negative outcomes [2]. Over the past 20 years, the syndemic approach has grown in impact and relevance for both global health and global mental health [3].

It is well recognized that mental and physical health conditions may co-occur and interact in ways that influence outcomes. For example, co-occurrence of depression and diabetes is known to lead to adverse effects on both morbidity and mortality [1]; depression has been associated with a 1.5-fold increase in mortality in people with diabetes [4]. A key difference between understanding the co-occurrence of conditions and the syndemic perspective is that the syndemic approach moves beyond comorbidity and considers the synergistic effects of the wider social, political and environmental contexts in terms of the factors which influence aetiology and prognosis, at both population and individual levels [5]. Using the example of depression and diabetes, a syndemic approach considers the circumstances under which these conditions interact. This could refer to socio economic factors that may be associated with depression and diabetes, such as poverty and exposure to trauma or violence, as well as the wider economic context such as trade policies promoting the production of highly processed, high calorie foods and also the health system itself in which these diseases are treated [1].

Of great significance in 2020 is the global Covid-19 pandemic and it is relevant to ask how using a syndemic approach can enhance our understanding and response to this global pandemic [6]. What are the relationships between cardiovascular and respiratory diseases, gender, ethnicity, socio-economic status, age and Covid-19? How do the health system and wider socio economic context in which Covid-19 is being managed influence outcomes [6]? Would a syndemic approach enhance our understanding and inform management and policy?

## IMPLICATIONS FOR RESEARCH AND CLINICAL PRACTICE

Taking a syndemic approach highlights these wider contexts which may be missed in patient-level clinical practice [1]. Despite the emphasis on personalized, holistic care, generic guidelines may at times lead to a "one size fits all" approach for patients. However, within any patient population there will be diversity in terms of social situation, ethnicity, age, financial circumstances, culture, political views, health beliefs, exposure to adverse events and a range of other factors. In health systems, there will be differences in terms of structure, service style, accessibility and wider policy, economic and environmental influences [1].

A number of vignettes have been published in which taking a syndemic approach influences clinical practice and increases the effectiveness of interventions [1]. For example, a "syndemic care system" is proposed for managing patients with diabetes and depression in South Africa. For this particular context, a community-based clinic structure is suggested that in addition to testing for single disorders, routinely provides screening for major comorbidities including mental disorders. This would enable formulation of a comorbidity profile and enhance provision of holistic care plans [1].

It is important to note that a syndemic approach does not necessarily have to lead to more complex multi-level interventions, which might seem unrealistic. Due to the synergistic nature of interacting factors, a syndemic approach suggests a single-component intervention may have scope to influence outcomes at multiple levels [5]. This is of particular relevance in contexts with limited resources, where affordability of multiple component interventions is low.

## IMPLICATIONS FOR GLOBAL MENTAL HEALTH

As well as in clinical practice, using a syndemic framework when considering wider public health initiatives can improve outcomes of whole population level interventions. Using a syndemic approach means rather than single disorders being considered one at a time, multiple disorders are considered together and the specific and shared wider context is explicitly taken into account [2]. For example, Brazil's Bolsa Familia Programme in 2003 distributed financial support to a quarter of the population in 2011, with the condition that children would go to school (where they would also receive food, vaccinations and growth monitoring) and women would attend postnatal services [2]. This intervention decreased poverty related malnutrition, diarrhoeal disease and overall mortality among children under five [2]. By addressing social inequality, this intervention benefited wider health outcomes due to the interactive nature of contributing factors.

Using the syndemic approach to develop mental health policy globally is crucial. The mental health "treatment gap" remains high in countries throughout the world and new initiatives are needed to address the increasing burden of mental disorders, especially in low- and middle- income countries, where resources are limited [7].

## CONCLUSION

The syndemic approach is of great relevance to enhancing the development of mental healthcare globally in terms of clinical practice, research and policy. In Russia, the syndemic framework has already begun to shape research priorities. Potential syndemics identified in Russia to date are directly related to mental healthcare, including the syndemic of "incarceration, injection drug use, poverty and alcohol abuse" [8] and "opioid addiction, HIV, hepatitis, tuberculosis, imprisonment and overdose" [9]. Further research into how a syndemic framework can enhance development of mental health care in Russia will build upon the strong background of integrated mental healthcare currently provided within polyclinics and dispensaries [10].

## Authors contribution

Sarah Jane Parry: reviewing publications on the theme of the article, article writing and editing. Sir Graham Thornicroft: reviewing publications on the theme of the article, designing article structure, article editing.

## Acknowledgements

Sir Graham Thornicroft is supported by the National Institute for Health Research (NIHR) Applied Research Collaboration South London at King's College London NHS Foundation Trust and by the NIHR Asset Global Health Unit award. The views expressed are those of the author(s) and not necessarily those of the NHS, the NIHR or the Department of Health and Social Care. GT also receives support from the National Institute of Mental Health of the National Institutes of Health under award number R01MH100470 (Cobalt study). GT is supported by the UK Medical Research Council in relation to the Emilia (MR/S001255/1) and Indigo Partnership (MR/R023697/1) awards.

## Conflict of interest

The authors declare no conflict of interest.

## Funding

Not applicable.

## Informed consent of patients

Not applicable.

## Compliance with principles of bioethics

Not applicable.
